# A Study of Corrosion-Grade Recognition on Metal Surfaces Based on Improved YOLOv8 Model

**DOI:** 10.3390/s25082630

**Published:** 2025-04-21

**Authors:** Hao Chen, Ying Cao, Shengxian Cao, Heng Piao

**Affiliations:** School of Automation Engineering, Northeast Electric Power University, Jilin 132012, China; 2202200610@neepu.edu.cn (H.C.); 2022303050209@neepu.edu.cn (Y.C.); piaoheng@neepu.edu.cn (H.P.)

**Keywords:** identification of metal corrosion status, YOLOv8, attention mechanism, data augmentation, dynamic learning rate adjustment

## Abstract

**Highlights:**

This paper is oriented towards the study of the corrosion state of typical equipment in a substation and establishes a model of a corrosion-state recognition algorithm for typical equipment in a substation based on an improved YOLOv8 model by introducing an attention mechanism for YOLOv8, designing a mixed-mixed sample data augmentation algorithm, and adopting a cosine annealing learning rate adjustment.

**What are the main findings?**

- An improved YOLOv8 model is proposed.
- The improved YOLOv8 model achieves significant performance improvements.

**What are the implications of the main findings?**

- The model provides an automated, efficient, and accurate solution for detecting metal equipment corrosion.
- The optimization strategies can serve as valuable references for other computer vision tasks, promoting advancements in related fields.

**Abstract:**

Typical metal equipment in substations is exposed to high-temperature, high-humidity, and high-salt environments for a long time, and surface corrosion is a serious threat to operational safety. Traditional manual inspection is limited by the complexity of the environment and subjective assessment errors, and there is an urgent need for a method that can quickly and accurately locate the corrosion area and assess the degree of corrosion. In this paper, based on YOLOv8, the feature extraction ability is improved by introducing the attention mechanism; a mixed-mixed-sample data augmentation algorithm is designed to increase the diversity of data; and a cosine annealing learning rate adjustment is adopted to improve the training efficiency. The corrosion process of metal materials is accelerated by a neutral salt spray test in order to collect corrosion samples at different stages and establish a dataset, and a model of a corrosion-state recognition algorithm for typical equipment in substations based on an improved YOLOv8 model is established. Finally, based on ablation experiments and comparison experiments, performance analyses of multiple algorithmic models are conducted for horizontal and vertical comparisons in order to verify the effectiveness of the improved method and the superiority of the models in this paper. The experiments verify that the improved model is comprehensively leading in multi-dimensional indicators: the mAP reaches 96.3% and the F1 score reaches 93.6%, which is significantly better than mainstream models such as Faster R-CNN, and provides a reliable technical solution for the intelligent inspection of substation equipment.

## 1. Introduction

The substation is an important part of the power system, in which the normal operation of metal equipment is critical to the stability and safety of the power system. In the substation environment, there are factors such as humidity, corrosive gases, and chemicals, and metal equipment is often affected by corrosion [1,2]. Corrosion not only reduces the performance and life of metal equipment, but also may lead to equipment failure, power loss, and even accidents. China’s annual economic losses due to rust and corrosion exceed CNY 2 trillion, accounting for 3.34% of the gross national product, of which the direct losses due to rust and corrosion in power transmission amount to CNY 79.4 billion. Therefore, the direct economic losses caused by corrosion are huge, and in order to prevent the problem of the corrosion of station equipment from becoming more serious, timely detection, assessment, and maintenance by station staff are required [3,4]. However, due to the complexity of the substation environment, this makes the inspection process difficult, which reduces the efficiency and accuracy of the inspection.

Currently, rust detection methods are mainly based on image processing techniques and deep learning techniques. In the traditional image processing field, researchers mainly achieve rust recognition by manually designing feature extraction rules. Nelson et al. [5] developed a wavelet-based denoising method using wavelet edge denoising detection to identify the potential rust region and the spatial distribution of rust, and generate a binary image for final corrosion classification. Liao et al. [6] constructed a three-level classification model based on HIS-RGB hybrid feature space, and the intelligent identification of steel structure corrosion was achieved by the multi-dimensional feature fusion of grey-scale hue percentage and the coefficient of variation combined with a least-squares support vector machine; however, this kind of method is constrained by the sensitivity of image quality and the problem of complex background interference, and there are obvious limitations in complex and multi-dimensional environmental scenes. With the breakthrough of deep learning technology, target detection algorithms provide a new paradigm for rust recognition. Tian [7] proposed a new method based on the combination of Faster-RCNN and HIS color space features for steel-plate rust detection and recognition, which can effectively improve the feature extraction capability and efficiency of neural networks. Guo et al. [8] constructed an improved architecture based on Faster-RCNN that significantly improves the detection accuracy of gold tool rust through the co-optimization of the ResNet-101 backbone network and an ROI feature enhancement mechanism. Katsamenis et al. [9] constructed a pixel-level rust localization system by innovatively adopting a U-Net semantic segmentation network supplemented with data projection optimization. Zhao Z et al. [10] integrated HSV color enhancement into the YOLOv7 framework, integrating HSV color enhancement, BiFPN multi-scale fusion, and a GSConv space preservation module to achieve the accurate capture of the corrosion features of an anti-vibration hammer. For the feature extraction problem of high-dimensional data, the data features can be strengthened by adding an attention module. Wang et al. [11] found that introducing the self-attention mechanism into neural networks can significantly improve the performance of neural networks. WOO et al. [12] firstly found that, for the feature maps in CNNs, a large amount of information exists inside the channels as well, and proposed a convolutional attention model. The model is able to obtain more comprehensive feature information by constructing spatial attention modules and channel attention modules and performing weighted aggregation. Compared with manual visual inspection, these methods can not only achieve more efficient data processing and analysis, saving time and manpower costs, but also achieve the precise positioning and accurate quantification of corrosion features, providing more refined corrosion assessment results [13].

YOLO [14,15,16,17] (You Only Look Once) is an important algorithm in the field of target detection in computer vision detection technology, and has the following advantages compared to other models: (i) fast speed, adapting to real-time system requirements; (ii) global vision, considering the overall contextual information with high accuracy; and (iii) simplicity, without the need for complex pipeline operations. It is an end-to-end training model. Among the versions of YOLO, YOLOv8 [18] is a more cutting-edge and advanced model that builds on the success of previous YOLO versions and introduces new features and improvements, with specific innovations including a new backbone network, a new Ancher-Free detection header, and a new loss function to further enhance performance and flexibility.

This paper is oriented towards the study of the corrosion state of typical equipment in substations. Based on the characteristics of the corrosion surface, such as color, texture, and shape, and in view of problems such as the high similarity of the characteristics of adjacent corrosion levels that are difficult to judge, the lack of datasets, and slow convergence speed, a model for a corrosion-state recognition algorithm for typical equipment in substations based on an improved YOLOv8 model is established. The model can automatically, efficiently, and accurately detect the corrosion state on the surface of metal equipment, which not only solves the problems of complexity and uncertainty existing in manual detection, but also provides a reference basis for subsequent maintenance work.

## 2. Materials and Methods

### 2.1. YOLOv8 Model Overview

**Figure 1:**
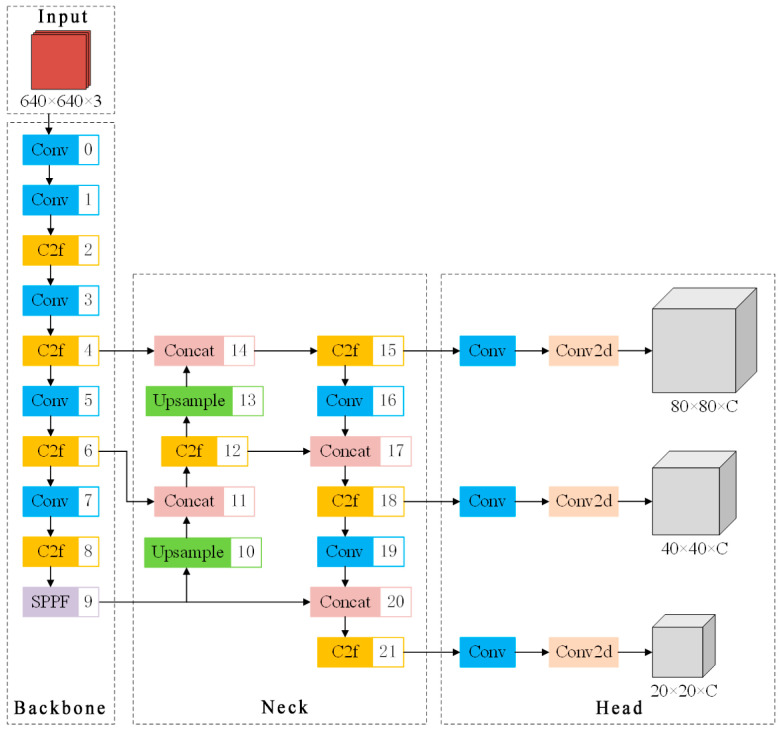
YOLOv8 network architecture diagram.

The YOLOv8 network structure is shown in Figure 1. The YOLOv8 network structure [19] consists of a backbone network, a neck network, and a head network. Its backbone network structure references the CSPDarknet structure, which includes five Conv modules, four C2f modules, and one SPPF module. Among them, the Conv modules are responsible for downsampling, the C2f modules are used to extract the feature information in the image, and the SPPF module enhances the efficiency and effectiveness of feature extraction through pooling operation.

YOLOv8′s neck network is responsible for further processing and the fusion of features from the backbone network, which adopts the idea of FPN + PAN [20] The main idea of FPN is to construct a pyramidal feature graph, which passes the semantic information from the higher layers to the lower layers through the top–down path to realize multi-scale feature fusion. FPN + PAN adds an additional bottom–up path, so that each layer not only receives information from the higher layer, but also obtains information from the lower layer, which improves the utilization of information from each layer.

**Figure 2:**
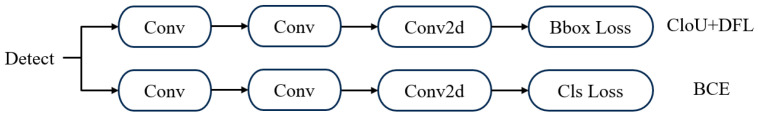
Decoupling head structure.

The head network of YOLOv8 introduces the decoupled head and Anchor-Free mechanism [21], which is responsible for the final target detection and classification tasks. Each scale of the decoupled head possesses an independent detector that is specifically responsible for predicting the bounding box on that scale, which can effectively improve the flexibility and computational efficiency of the network model, and the structure of the decoupled head is shown in Figure 2. Before entering the regression and classification branch, the feature map is preprocessed by two Conv modules and a 2D convolutional layer. In the regression branch, the position offset between the predicted frame and the real frame is first calculated, and then the offset is used to calculate the loss value, and finally a four-dimensional vector is output, which represents the upper-left and lower-right coordinates of the target frame. In the classification branch, each Anchor-Free extracted candidate box undergoes RoI pooling and convolution operations to generate a tensor of classifier outputs, where the value of each position indicates the probability that the candidate box belongs to each category. Finally, the final detection results are filtered using a non-great suppression method.

### 2.2. Model Improvement Methods

#### 2.2.1. Attention Mechanism

**Figure 3:**
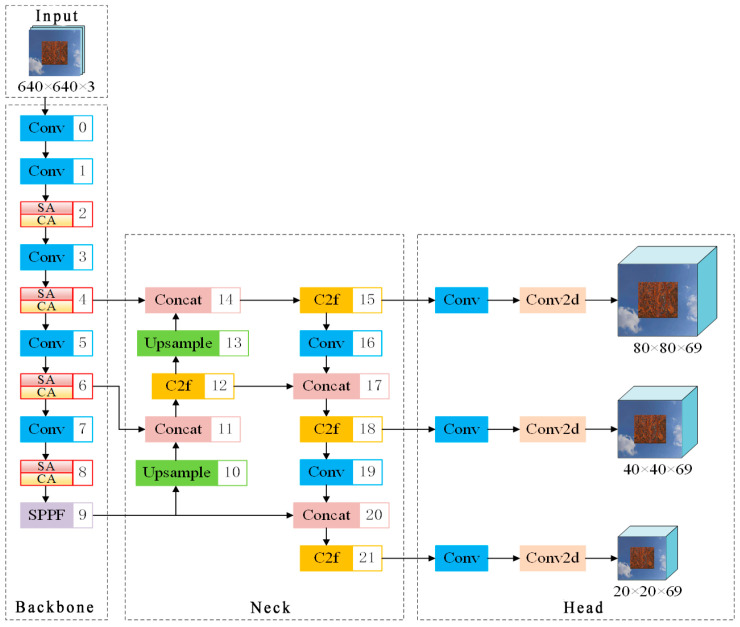
Improved YOLOv8 model structure.

In order to realize adaptive context extraction, balance the intensity of multi-order interactions, and improve the extraction ability of rust detail features, this paper replaces the C2f module in the YOLOv8 backbone network with the spatial aggregation module of the multi-order gated aggregation network and the channel aggregation module, and the structure of the improved YOLOv8 model is shown in Figure 3. The spatial aggregation module encapsulates local perception and gating context aggregation together, forcing the aggregation of features that interact with contexts that are ignored through parallel processing. From a channel perspective, existing approaches tend to produce a lot of redundant information, while the channel aggregation module adaptively forces the network to encode expressive interactions that would otherwise be ignored. Intuitively, it performs the channel reassignment of inputs, outperforming popular existing methods with much lower computational overhead.

**Figure 4:**
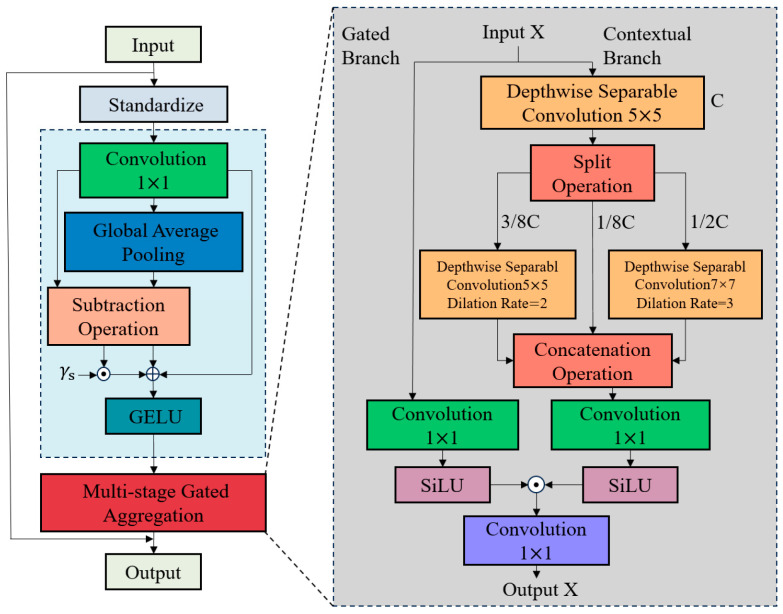
Space aggregation module (SA).

The spatial aggregation module (SA) is used to force the network to encode otherwise ignored expressive interactions and useful information, thus enabling the model to capture more middle-order interactions. The structure of the spatial aggregation module is shown in Figure 4, which consists of two tandem components:

(1) $$Z=X+Moga\left(FD\left(Norm\left(X\right)\right)\right)$$

where *FD*(.) denotes the feature decomposition module and *Moga*(.) denotes the multi-order gated aggregation module, including gating and context branching. As a pure convolutional neural network structure, multi-order features are extracted by static and adaptive local sensing. There are two complementary counterparts, i.e., fine-grained local texture (low-order) and complex global shape (middle-order), which are instantiated by Conv_1×1_(.) and GAP(.), respectively. To force the network to focus on balancing the multi-order interactions, *FD*(.) is used to adaptively exclude trivial interactions, which are defined as follows:

(2) $$Y=Conv_{1\times 1}\left(X\right)$$

(3) $$Z=GELU(Y+\gamma_{s}e(Y\text{-}GAP(Y)))$$

where $\gamma_{s}\in R^{C\times 1}$ denotes the scaling factor initialized to zero. *FD*(.) increases spatial feature diversity by re-weighting the complementary interaction component *Y-GAP(Y)*. Deep convolution is then used to encode the multi-order features in the context branch of *Moga*(.), employing three different deep convolutional layers with parallel dilation ratios $d\in \left\{1,2,3\right\}$ to capture low-, middle-, and high-order interactions.

Given the input feature $X\in R^{C\times \mathrm{HW}}$, ${\mathrm{DW}}_{5\times 5,d=1}$ is first applied to extract the low-order features; then, the output is decomposed along the channel dimensions into $X_{l}\in R^{C_{l}\times \mathrm{HW}}$, $X_{m}\in R^{C_{m}\times \mathrm{HW}}$, and $X_{h}\in R^{C_{h}\times \mathrm{HW}}$, where $C_{l}+C_{m}+C_{h}=C$; after that, $X_{m}$ and $X_{h}$ are assigned to ${\mathrm{DW}}_{5\times 5,d=2}$ and ${\mathrm{DW}}_{7\times 7,d=3}$, respectively, while $X_{l}$ serves as a constant mapping; finally, the outputs of $X_{l}$, $X_{m}$, and $X_{h}$ are connected to form a multi-order context: $Y_{C}=\mathrm{Concat}(Y_{l,1:C_{l}},Y_{m},Y_{h})$.

In order to adaptively aggregate the features extracted from the context branch, the SiLU activation function is used in the gated branch, i.e., x·sigmoid(x). The output of FD(.) is taken as an input and is represented by the following equation:

(4) $$Z=SiLU(Conv_{1\times 1}(X))⊙SiLU(Conv_{1\times 1}(Y_{C}))$$

**Figure 5:**
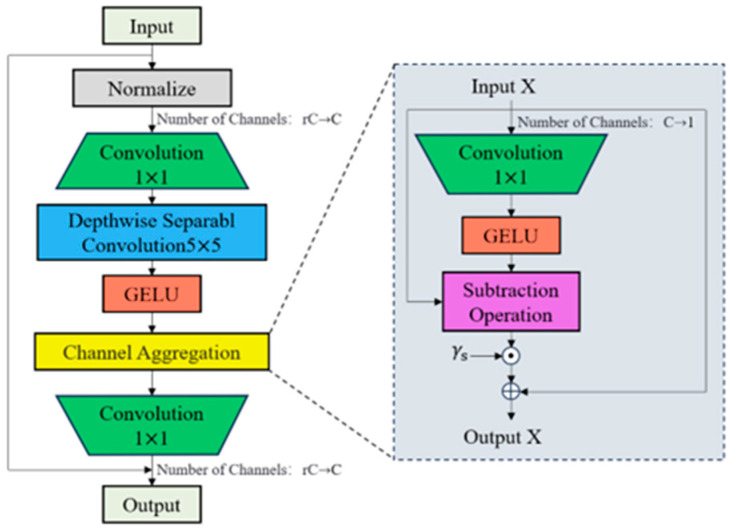
Channel aggregation module.

Due to the redundancy of information between channels, ordinary multi-layer perceptrons require multiple parameters to achieve the expected performance, resulting in low computational efficiency. To solve this problem, a lightweight channel aggregation module CA(.) is introduced after the spatial aggregation module for adaptively redistributing the channel features in the high-dimensional hidden space, and the structure of the channel aggregation module is shown in Figure 5. The channel aggregation module is represented by the following equation:

(5) $$Y=GELU\left(DW_{3\times 3}\left(Conv_{1\times 1}\left(Norm\left(X\right)\right)\right)\right)$$

(6) $$Z=Conv\left(CA\left(Y\right)\right)+X$$

Specifically, *CA*(.) is realized by the channel reduction projection $W_{r}:\;R^{C\times \mathrm{HW}}\to R^{1\times \mathrm{HW}}$, and uses the *GELU* activation function to collect and redistribute channel information:

(7) $$CA(X)=X+\gamma_{c}⊙(X-GELU(XW_{r}))$$

where $\gamma_{c}$ is a channel scaling factor initialized to 0 that redistributes channel features through complementary interactions *(X−GELU(XW_r_))*, thus enhancing the initially neglected game–theoretic interactions.

#### 2.2.2. Data Augmentation

**Figure 6:**
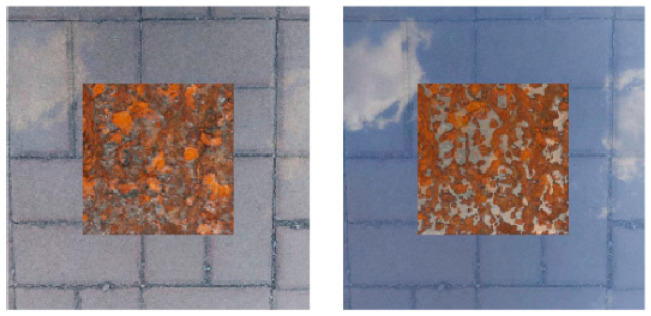
Two randomly selected mixed-sample images.

**Figure 7:**
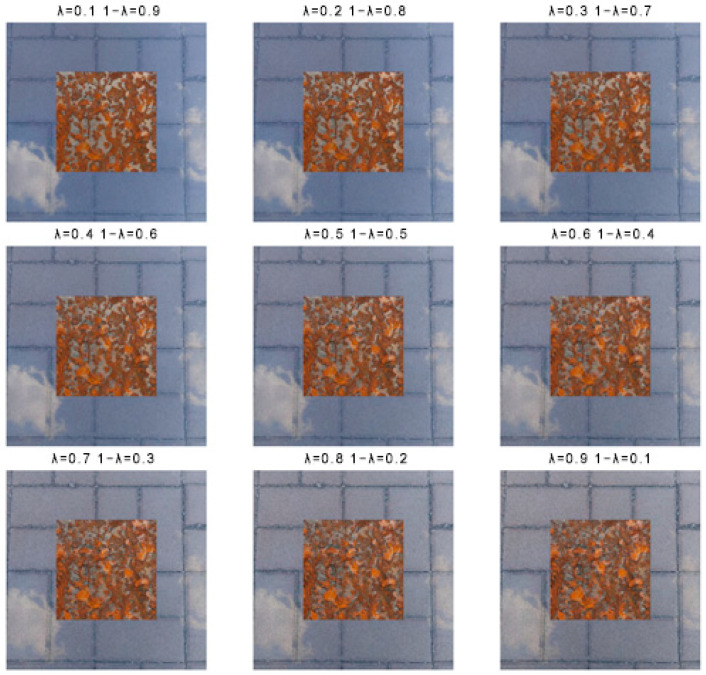
Images of the two mixed samples after they have been scaled up again.

The main aspects of YOLOv8 in terms of data enhancement include stochastic affine transformation [22], mixed-sample data [23], flipping [24], zooming [25], warping [26], color gamut transformation [27], and mosaic splicing [28], introducing the operation of turning off mosaic splicing for the last 10 rounds of training, as proposed in YOLOX. Among them, the core idea of mixed-sample data augmentation is to linearly interpolate two images in a certain proportion, and at the same time perform the same linear interpolation on their labels. In this paper, on the basis of mixed-sample data augmentation, the two randomly selected mixed samples are again subjected to mixed data augmentation, and a mixed-mixed-sample data augmentation method is proposed. The formula of the mixed-mixed-sample data augmentation method is as follows:

(8) $$\tilde{x}=\lambda x_{i}+(1-\lambda )x_{j}$$

(9) $$\tilde{y}=\lambda y_{i}+(1-\lambda )y_{j}$$

where *x_i_* and *x_j_* are two randomly selected mixed samples from two images after mixed-sample data augmentation, *y_i_* and *y_j_* are the corresponding labels, and λ is a random number within [0, 1], set to 0.1 to 0.9. Two randomly selected mixed-sample images are shown in Figure 6; these two mixed samples are again proportionally blended, as shown in Figure 7. Then, two randomly selected mixed-sample images, which are again proportionally blended via mixed data augmentation, are again blended proportionally.

#### 2.2.3. Learning Rate Adjustment

In order to improve the accuracy and convergence speed of the network model on the rust classification task, the cosine annealing learning rate adjustment is chosen in this paper. The mainstream learning rate adjustments are broadly categorized into fixed-step decay, multi-step decay [29], and cosine annealing decay [30]. The fixed-step decay decays the learning rate by decaying it by a certain percentage after each training cycle or a specific number of steps. Multi-step decay allows the learning rate to be adjusted at multiple specified time points, whereas the cosine annealing decay adjusts the learning rate by introducing a cosine function to undergo a periodic change from slow decline to acceleration to slow decline, which helps the learning rate to quickly recover to the initial value after reaching the minimum point and continuously search for the global optimal solution.

The cosine annealing decay strategy, combined with the introduction of the attention mechanism and the mixed-mixed-sample data augmentation method, synergistically improves the model’s performance effectively. The spatial aggregation module filters multi-scale contextual features through the gating mechanism, while the channel aggregation module strengthens the key frequency band response, suppresses the redundant channel interference, and improves the interactions between middle-order and high-order features. The periodic learning rate fluctuation of the cosine annealing attenuation strategy enables the model to more accurately capture the subtle differences. The mixed-mixed-sample data augmentation method significantly improves the complexity of the data distribution, and the conventional learning rate decay is prone to triggering gradient oscillations under the data. The cosine annealing decay strategy enables the model to adapt to the complex distribution asymptotically in the dynamic learning rate and dynamically adapt to the changes in the feature distribution through the periodic learning rate reset. The cosine annealing decay strategy is formulated as follows:

(10) $$\eta_{t}=\eta_{\min}+0.5(\eta_{\max}-\eta_{\min})(1+\cos(T_{cur}\pi /T_{\max}))$$

where *η*_max_ and *η*_min_ denote the range of the learning rate, and *T*_cur_ and *T*_max_ denote the current and total number of iterations, respectively.

### 2.3. Dataset Production

**Figure 8:**
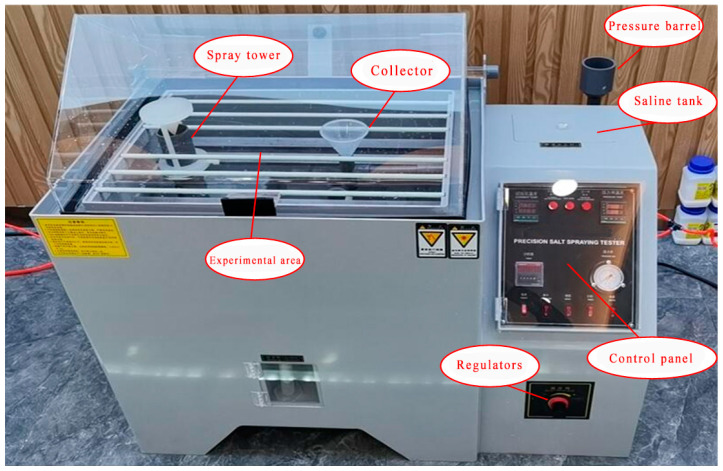
Salt spray test chamber.

In this paper, a neutral salt spray accelerated corrosion test [31] was conducted on nine types of carbon steel plates using a GM-60 salt spray test chamber to construct a corrosion image dataset. The salt spray test chamber simulates the corrosive environment by artificially constructing a high-concentration salt spray environment, accelerating the corrosion process of the metal surface in order to quickly obtain the image data of different corrosion stages and accelerate the experimental process, and the salt spray test chamber is shown in Figure 8. The test samples are nine types of carbon-steel-plate materials, including 10#, 20#, 25#, 30#, 35#, 45#, Q195, Q235, and Q345. The 10#–45# series has excellent machinability, and is mainly used for the manufacture of bolts, connecting rods, and other connecting parts of the substation; Q195 is used for thin-walled components, such as instrument housings and cable troughs by virtue of its good weldability; Q235 is widely used as a general carbon structural steel in transmission towers, equipment brackets, and other load-bearing structures; and Q345 is used as a low-alloy high-strength steel, suitable for electrical equipment and other high-performance components. The chemical composition of each model is shown in Table 1 and Table 2, and the difference in composition directly affects the corrosion resistance of the material and the corrosion morphology characteristics.

Accelerated corrosion tests were performed on nine types of carbon steel plates (2 pieces of each type, size 100 × 100 × 3 mm) during the test. The tests were conducted in 12 h cycles with neutral salt spray, and 10 cycles were executed in total (120 h in total). At the end of each cycle, the samples were removed by demisting, allowed to dry for 0.5–1 h, rinsed of surface salt residues, and the color temperature (5500 K ± 200) and illuminance (800 Lux ± 5%) were fixed by means of an industrial-grade filler lamp. The corrosion pattern was recorded using an equipped high-definition camera, followed by a restart of the test. The test samples were classified into five levels according to the corrosion duration: level 1 for 12 and 24 h, level 2 for 36 and 48 h, level 3 for 60 and 72 h, level 4 for 84 and 96 h, and level 5 for 108 and 120 h. In total, 720 standardized corrosion sample images were obtained by background cropping and the cross-segmentation of the original image.

**Figure 9:**
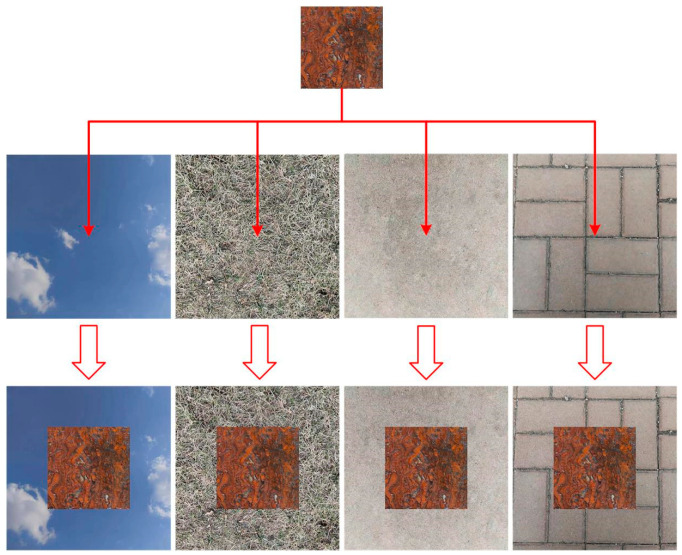
Four different background images and rust images fused with the corresponding backgrounds.

In order to enhance the diversity and complexity of the dataset, in this paper, the original 720 cropped images are synthesized with four types of substation live backgrounds, namely, blue sky, lawn, pavement, and tile, to generate 2880 multi-scene images (576 images per class). The four different background images and the rusted images fused with the corresponding backgrounds are shown in Figure 9. In deep learning tasks, the model relies on a large amount of data to improve the generalization ability, the training set is used to train the model parameters, the validation set is used for hyper-parameter adjustment, and the test set is used for the final evaluation. The images in this paper are annotated by LabelImg [32] and then divided into the training set (2304), the validation set (288), and the test set (288) according to the ratio of 8:1:1, and constructed to cover all grades and all scenes of the ‘Metal corrosion-grade classification dataset’. Notably, 80% of the training data can fully train the model parameters, reducing underfitting due to insufficient data. The validation set and test set usually need to cover dozens of samples for each category, at least. This paper has a balanced distribution of data categories: the validation set and the test set each accounted for 10%. The number of 288 datapoints ensures that each category has about 56 samples to meet the statistical significance threshold and the basic needs of model tuning and performance evaluation. At the same time, the ratio of 8:1:1 reduces the amount of validation and test data, which can speed up the model iteration speed, and is suitable for scenarios with limited arithmetic power or the need for rapid experimentation.

**Figure 10:**
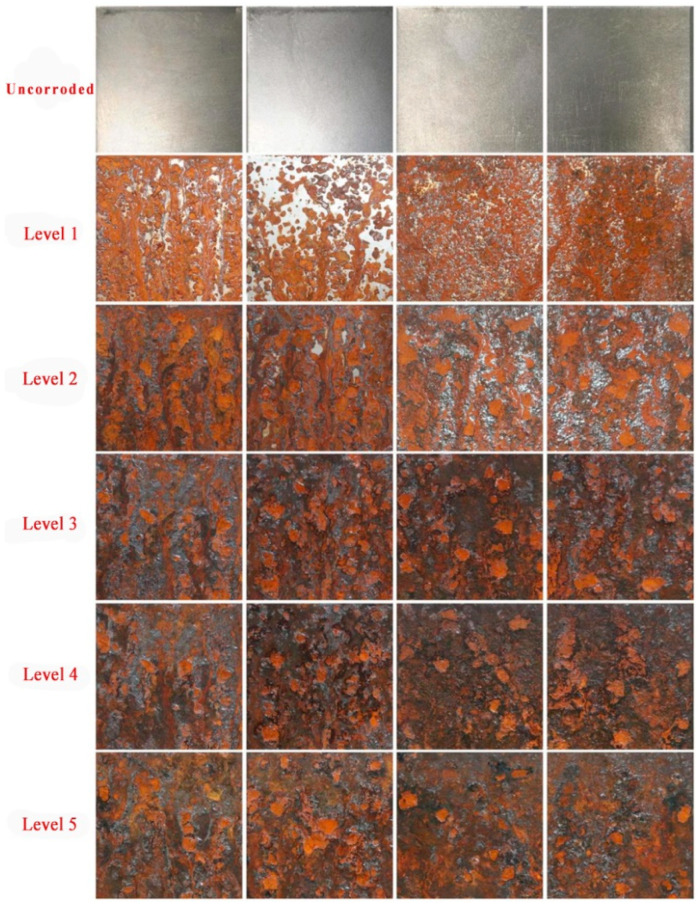
Part of the initial sample image and the corresponding five levels of rust sample images.

Part of the initial sample image and the corresponding images of the five levels of corrosion are shown in Figure 10. Through Figure 10, sample comparison can be seen in the corrosion process characteristics: level 1 shows a localized un-rusted area—the un-rusted area decreases as time increases; level 2 basically covers the orange-red rust layer with a residual sporadic clean area; level 3 forms a clear and rough texture accompanied by oxide flaking; level 4 has dark corrosion craters and black spots; and level 5 presents a large area of black oxide and metal matrix exposure. There is a significant color gradient (orange-red→dark brown→black) and progressive correlation with structural deterioration (pitting→spalling→exposed substrate) among the levels.

### 2.4. Experimental Environment and Evaluation Indicators

In this paper, we adopt the zero pre-training strategy. The experimental environment is configured with the Intel Xeon E5-2698v4 CPU + NVIDIA RTX 4070 (12 GB video memory) hardware platform, relying on the PyTorch 1.12 framework to realize the model development, and is accelerated by CUDA 11.6 parallel computation to meet the demands of large-scale data iteration. The hyperparameter settings are shown in Table 3.

In order to comprehensively evaluate the recognition ability of different network models in the task of metal corrosion-grade classification, this paper adopts precision, recall, F1 score, and mean average precision mean (mAP) [33] as the core evaluation metrics. The evaluation metrics involved in this paper are formulated as follows:

(11) P=TP/(TP+FP)

(12) R=TP/(TP+FN)

(13) F1=2×(P×R)/(P+R)

(14) $$AP=\int_{0}^{1}P\left(R\right)dr$$

(15) $$mAP=(\sum_{i=1}^{N}AP_{i})/N$$

where *TP* is the true case, i.e., the number of samples that are actually positive categories and correctly predicted as positive by the model; *FP* is the false-positive case, i.e., the number of samples that are actually negative categories but incorrectly predicted as positive by the model; *FN* is the false-negative case, i.e., the number of samples that are actually positive categories but incorrectly predicted as negative by the model; *P* is precision; *R* is recall rate; *N* is the number of categories; and *AP_i_* is the average precision of the ith category.

## 3. Results

### 3.1. YOLOv8 Model Ablation Experiment

In order to analyze the impact of each improvement on the YOLOv8 network model, this paper conducts an ablation experiment, which takes the original YOLOv8 model as the baseline and adds three improvement methods step by step: the model with only the spatial aggregation modules and channel aggregation modules added is called YOLOv8-1; the model with the mixed-mixed-sample data augmentation algorithm added on top of YOLOv8-1 is called YOLOv8-2; YOLOv8-1 and YOLOv8-2 use a fixed learning rate strategy, setting the learning rate to 0.01; and the model with the cosine annealing learning rate adjustment added on top of YOLOv8-2 is called YOLOv8-3, i.e., the final model with all the improvement methods added. The results of the ablation experiment are shown in Table 4.

**Figure 11:**
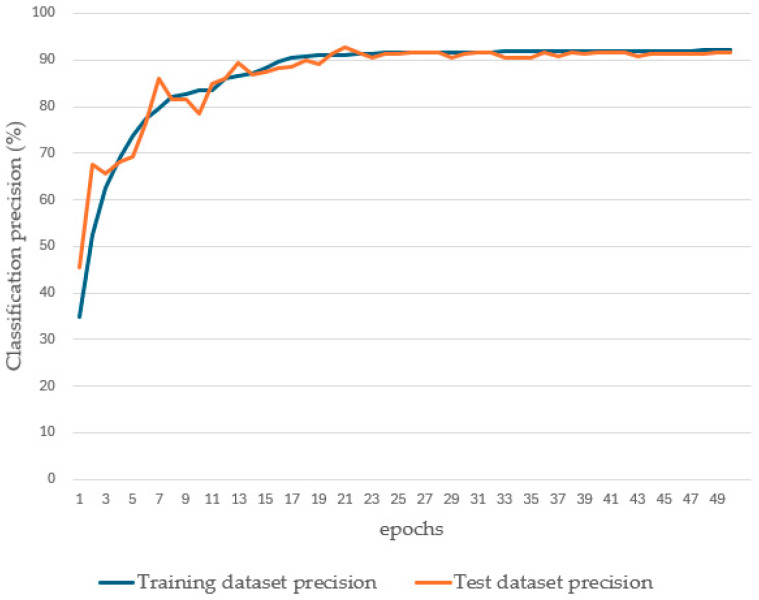
YOLOV8-3 precision data chart.

**Figure 12:**
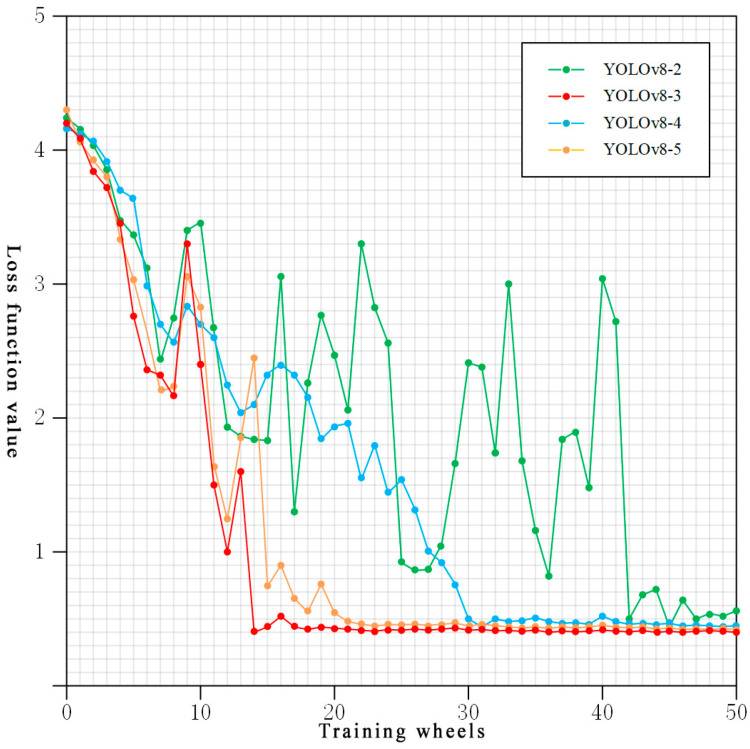
Comparison of loss function values for different learning rate adjustments.

The original YOLOv8 model performed as follows in all recognized categories: precision of 80.3%, recall of 83.8%, F1 score of 82%, and mAP of 90.3%. However, the recall is lower, at 70.2%, for recognizing rust level 3, and the precision is lower, at 65.4% and 77.7%, for recognizing rust levels 4 and 5, respectively. The overall performance of the YOLOv8-1 model improves significantly: in all recognition categories, the precision improves by 8.3%, the recall improves by 7.3%, the F1 scores improves by 7.9%, and the mAP improves by 2.6%. Recall improved by 15.4% in recognizing rust class 3, and precision improved by 12.1% and 7.4% in recognizing rust classes 4 and 5, respectively. This is attributed to the spatial aggregation modules and channel aggregation modules that enhance the model’s middle-order interaction capability, improve the focus on the most relevant features, and suppress the interference of irrelevant and weakly relevant information. The overall performance of the YOLOv8-2 model is further improved based on that of the YOLOv8-1 model: the precision is improved by 2.8%, the recall by 2.8%, the F1 score by 2.7%, and the mAP by 2.7% for all the recognition categories. Recall further improved to a high level of 90.5% when identifying rust class 3, and precision improved to 82.4% and 89.3% when identifying rust classes 4 and 5, respectively, also reaching a high level. A potential explanation is that the mixed-mixed-sample data augmentation algorithm increases the diversity of the training data by generating more samples and improves the generalization ability and robustness of the model with the help of the spatial aggregation module and the channel aggregation module. Eventually, the YOLOv8-3 model, with the introduction of the cosine annealing learning rate adjustment, performs as follows in all recognition categories: the precision is 92%, the recall is 95%, the F1 score is 93.6%, and the mAP is 96.3%. After 20 rounds of training, the training set accuracy and the test set accuracy both converge to a stable value of 92%. The specific data situation is shown in Figure 11, which indicates that the model not only understands the training data well, but also can generalize the learned laws to the real-world environment with a good generalization performance. Although the overall performance improvement of the YOLOv8-3 model over the YOLOv8-2 model is small in terms of evaluation metrics, the YOLOv8-3 model converges significantly faster than the YOLOv8-2 model in terms of the loss function, and a comparison of the loss function values for different learning rate adjustments is shown in Figure 12. In summary, each of these improvements improves the overall performance of the model, allowing the model to more accurately identify the degree of rusting on metal surfaces.

In order to further test the effect of different learning rate adjustments on the model, this paper also compares the fixed-step decay and the multi-step decay, and demonstrates the advantage of choosing the cosine annealing decay by observing the change in loss function values on the validation set. The model that adds a fixed-step decay to YOLOv8-2 is called YOLOv8-4, and the model that adds a multi-step decay is called YOLOv8-5. As shown in Figure 12, the loss function value of the YOLOv8-2 model fluctuates greatly throughout the training process and the fluctuation lasts for a long period of time, and there are even small fluctuations after 42 rounds of training; the YOLOv8-4 model fluctuates less, but converges more slowly, and stabilizes only after about 30 rounds of training; the YOLOv8-5 model fluctuates more in the pre-training, but converges rapidly after about 15 rounds of training and stabilizes at around 20 rounds of training. The loss function value of the YOLOv8-3 model fluctuates more in the pre-training as well, but converges rapidly after about 14 rounds of training and stabilizes, and its improvement effect is better than that of the YOLOv8-5 model. The experimental data show that the synergistic effect of the cosine annealing decay strategy, the attention mechanism, and the mixed-mixed-sample data augmentation method improves the model performance compared to other learning rate adjustment strategies, which enables the model to capture subtle differences more accurately and dynamically adapt to the changes in the feature distribution, improving the accuracy and convergence speed of the model on the rust classification task.

### 3.2. Comparison Experiment of Different Detection Models

**Figure 13:**
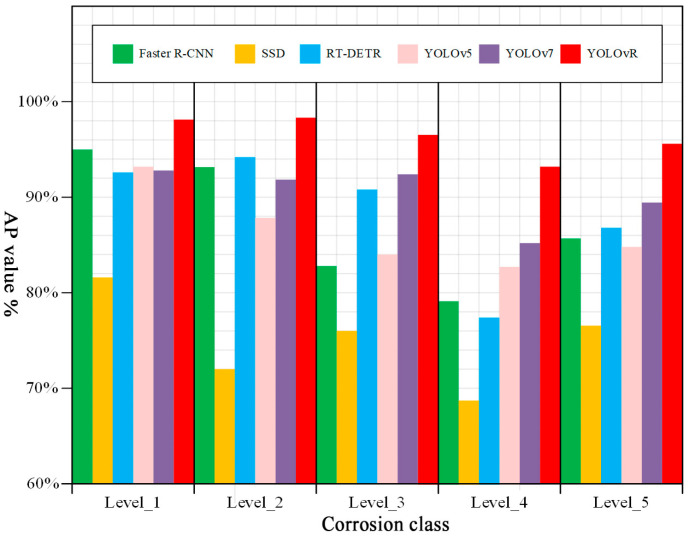
Comparison of average accuracy values of five rust levels for different models.

In order to verify the superiority of the improved models in the rust-grade classification task, five popular target detection algorithms, Faster R-CNN [34], SSD [35], RT-DETR [36], YOLOv5, and YOLOv7 [37] are selected in this paper for a comparative experiment. All models are trained and tested on the same dataset with the same hyperparameters, and the results of the comparison experiment are shown in Table 5. The comparison of the average accuracy values of the different models for the five rust levels is shown in Figure 13. The results show that the improved YOLOv8 model studied in this paper outperforms other mainstream models both in the four metrics of precision, recall, mAP, and F1 score, as well as in the AP values of the five rust levels.

**Figure 14:**
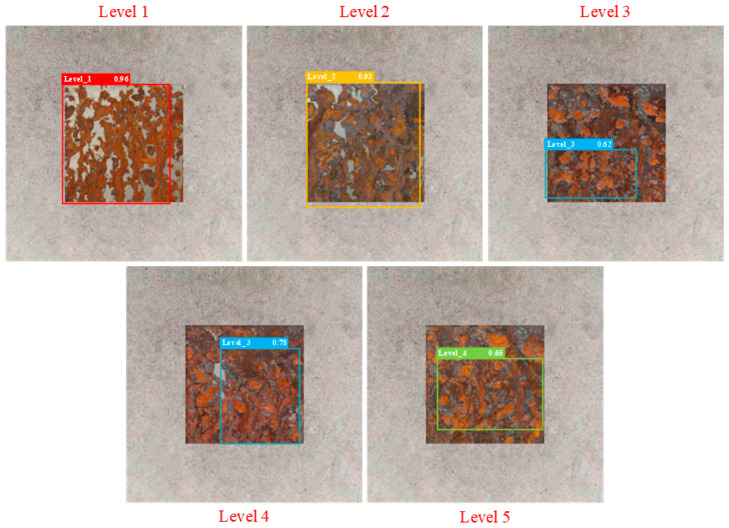
Faster R-CNN.

**Figure 15:**
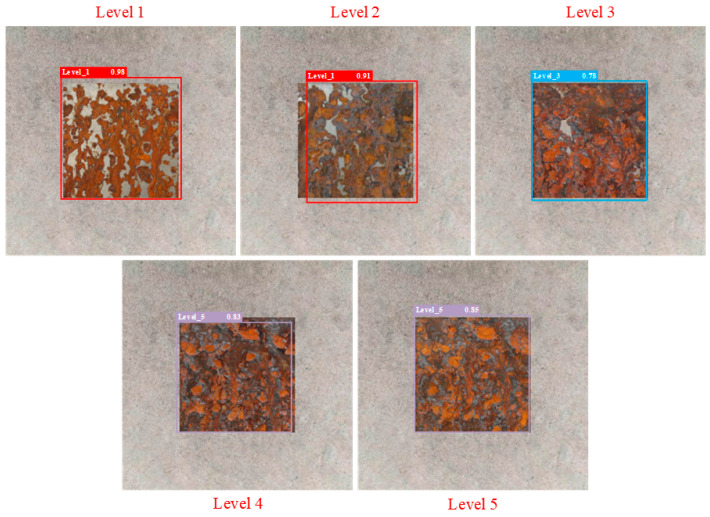
SSD.

**Figure 16:**
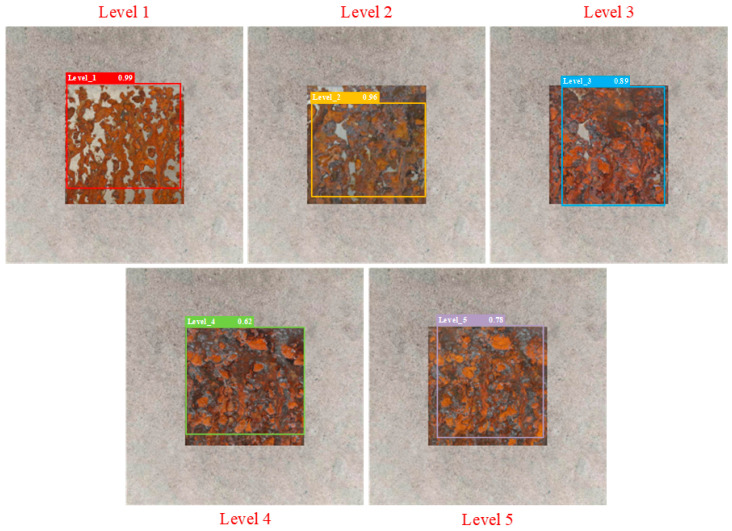
RT-DETR.

**Figure 17:**
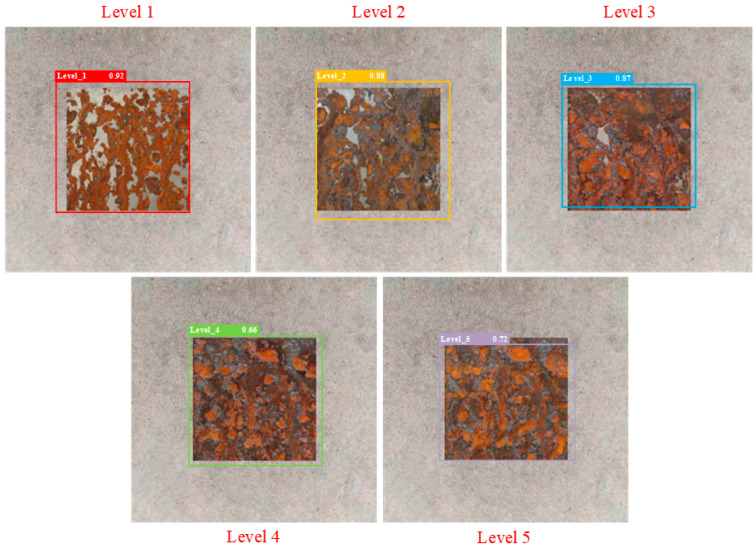
YOLOv5.

**Figure 18:**
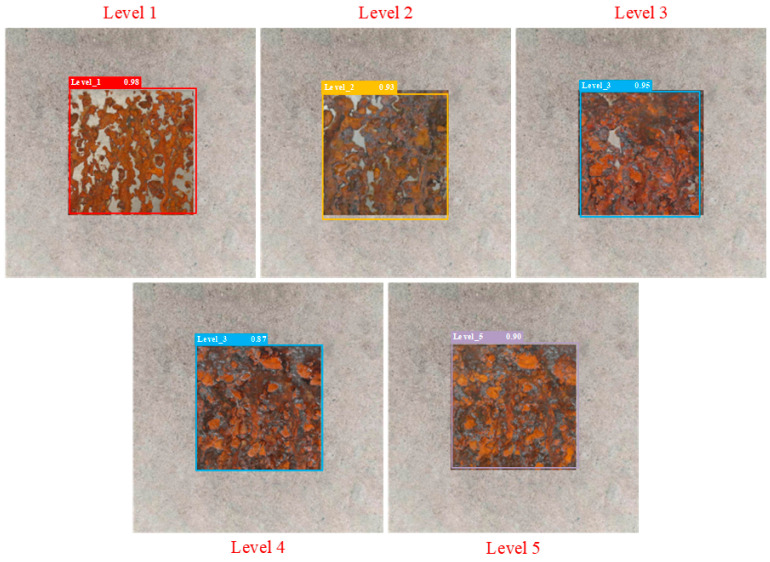
YOLOv7.

**Figure 19:**
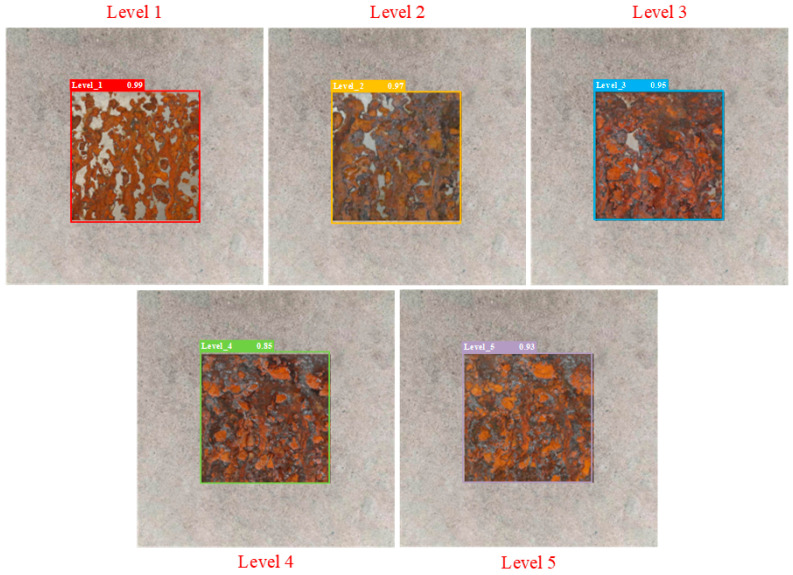
Improved YOLOv8 model.

The partial test results of different detection models are shown in Figure 14, Figure 15, Figure 16, Figure 17, Figure 18 and Figure 19, where each model outputs only the final classification result for a particular image, with the classification result, detection frame, and corresponding confidence score labelled in the image. The confidence score consists of the detection frame score and the target classification score output by the network, which is used to indicate the recognition rate of the rusted samples. In order to demonstrate the testing effect of different detection models on different rust levels, we recorded the confidence scores of different detection models for all rust classes in different images, and the specific data are shown in Table 6. Observing the detection frame positions and confidence scores outputted by each model, it is found that except for the improved model in this paper, the other five models have the problems of inaccurate positioning, low recognition rate, and rust level misdetection. Among them, the Faster R-CNN model has the problem of misdetection and the under-coverage of detection frames; SSD has cross-level misclassification; RT-DETR has the under-coverage of detection frames, although it classifies accurately; YOLOv5 exhibits boundary overflow and has a low recognition rate for high grades; and YOLOv7 has neighbor confusion (level 4 is misclassified as level 3). Only the improved model in this paper can not only accurately locate the rusted area, but also correctly identify rusted samples of five grades, and all detection frames have high confidence scores.

## 4. Discussion

In this paper, for the actual needs of substation metal equipment corrosion-state detection, a metal surface corrosion-grade identification method based on an improved YOLOv8 model is proposed, a typical substation equipment corrosion-state identification model is established, and ablation experiments and comparison experiments are carried out. The conclusions are as follows:

(1) In this paper, an attention mechanism is introduced to YOLOv8, and the C2f module in the backbone network is replaced by the spatial aggregation module of the multistage gated aggregation network and the channel aggregation module, which enhances the model’s intermediate-order interaction capability, improves the attention given to the most relevant features, and suppresses the interference of irrelevant and weakly relevant information, solving the problem of the high similarity of features of neighboring rusting levels. A mixed-mixed-sample data augmentation algorithm was designed to enhance the diversity and complexity of the dataset by generating more samples, which effectively improved the model’s adaptability to complex backgrounds and classification robustness. The cosine annealing learning rate adjustment is adopted to dynamically adjust the learning rate based on the cosine function curve, which improves the accuracy and convergence speed of the model on the rust classification task.

(2) The improved YOLOv8 model improves 11.7%, 11.2%, 11.6%, and 6% in terms of precision, recall, F1 score, and mAP, respectively, compared with the original model, and significantly outperforms other mainstream detection models, with excellent performance on the higher-order classification task for rust grades 3–5 and high localization accuracy of the detection frame.

The improved YOLOv8 model performs well in the task of identifying the corrosion level of metal surfaces, and is able to automatically, efficiently, and accurately detect the corrosion status of metal equipment surfaces, solving the complexity and uncertainty of manual detection and providing a reference basis for the subsequent maintenance of substation equipment.

## Figures and Tables

**Table 1:** Chemical composition of nine types of carbon steel plates (1).

Chemical Composition (%)	C	Si	Mn	P	S
10#	0.07–0.13	0.17–0.37	0.35–0.65	≤0.035	≤0.030
20#	0.17–0.23	0.17–0.37	0.35–0.65	≤0.035	≤0.030
25#	0.22–0.29	0.17–0.37	0.50–0.80	≤0.035	≤0.035
30#	0.27–0.35	0.17–0.37	0.50–0.80	≤0.035	≤0.035
35#	0.32–0.39	0.17–0.37	0.50–0.80	≤0.035	≤0.030
45#	0.42–0.50	0.17–0.37	0.50–0.80	≤0.035	≤0.035
Q195	0.06–0.12	≤0.30	0.25–0.50	≤0.050	≤0.045
Q235	0.12–0.22	≤0.30	0.30–0.80	0.035–0.045	0.035–0.045
Q345	≤0.20	≤0.55	≤1.70	0.025–0.045	0.025–0.045

**Table 2:** Chemical composition of nine types of carbon steel plates (2).

Chemical Composition (%)	Cr	Ni	Cu	V	Al
10#	≤0.15	≤0.30	≤0.25	\	\
20#	≤0.20	≤0.30	≤0.25	\	\
25#	≤0.20	≤0.30	≤0.25	\	\
30#	≤0.25	≤0.25	≤0.25	\	\
35#	≤0.20	≤0.30	≤0.25	\	\
45#	≤0.20	≤0.30	≤0.25	\	\
Q195	≤0.03	≤0.03	≤0.03	\	\
Q235	\	\	\	\	\
Q345	\	\	\	0.02–0.15	0/≥ 0.015

**Table 3:** Hyperparameter settings.

Hyperparameter	Value
Optimizer	SGD
Initial learning rate	0.01
Weight decay factor	0.0005
Momentum fixed value	0.937
Batch size	64
Training wheels	50
Input size	640 × 640

**Table 4:** Results of ablation experiment.

Model	Corrosion Class	Precision	Recall	F1 Score	mAP
YOLOv8	all	80.3%	83.8%	82%	90.3%
	Level 1	83.6%	92.1%	87.6%	93.6%
	Level 2	88.5%	84.3%	86.5%	95.0%
	Level 3	86.3%	70.2%	77.4%	88.9%
	Level 4	65.4%	88.9%	75.4%	84.2%
	Level 5	77.7%	83.5%	80.4%	89.7%
YOLOv8-1	all	88.6%	91.1%	89.9%	92.9%
	Level 1	91.4%	93.8%	92.6%	95.1%
	Level 2	96.7%	97.3%	97%	97.2%
	Level 3	92.2%	85.6%	88.8%	93.4%
	Level 4	77.5%	92.2%	84.2%	86.3%
	Level 5	85.1%	86.7%	86%	92.3%
YOLOv8-2	all	91.4%	93.9%	92.6%	95.4%
	Level 1	94.8%	95.2%	95%	97.6%
	Level 2	96.9%	99.3%	98.3%	97.8%
	Level 3	93.6%	90.5%	92.1%	95.2%
	Level 4	82.4%	96%	88.7%	91.8%
	Level 5	89.3%	88.6%	89%	94.4%
YOLOv8-3	all	92%	95%	93.6%	96.3%
	Level 1	96.1%	95.3%	95.6%	98.2%
	Level 2	97.2%	99.8%	98.5%	98.3%
	Level 3	94.1%	93.4%	93.7%	96.5%
	Level 4	83.3%	96.8%	89.5%	92.7%
	Level 5	89.5%	89.5%	89.5%	95.6%

**Table 5:** Comparative experimental results.

Model	Precision	Recall	mAP	F1 Score	FPS
Faster R-CNN	82.9%	76.8%	87.2%	79.7%	42.24
SSD	62.3%	78.1%	74%	69.2%	51.73
RT-DETR	79.6%	81.5%	88.4%	80.1%	48.86
YOLOv5	71.3%	89.3%	86.7%	79.3%	53.62
YOLOv7	75.5%	87.8%	90.7%	81.3%	56.08
Improved YOLOv8	92%	95%	96.3%	93.6%	51.12

**Table 6:** Confidence scores of different detection models for all rust levels.

Model	Corrosion Class	Level 1 Confidence Score	Level 2 Confidence Score	Level 3 Confidence Score	Level 4 Confidence Score	Level 5 Confidence Score
Faster R-CNN	Level 1	0.96	0.86	0.63	0.56	0.52
	Level 2	0.89	0.93	0.76	0.62	0.57
	Level 3	0.57	0.65	0.79	0.81	0.71
	Level 4	0.52	0.57	0.78	0.7	0.62
	Level 5	0.43	0.42	0.51	0.65	0.59
SSD	Level 1	0.98	0.89	0.81	0.65	0.61
	Level 2	0.91	0.86	0.82	0.71	0.56
	Level 3	0.61	0.68	0.78	0.7	0.63
	Level 4	0.42	0.39	0.65	0.72	0.83
	Level 5	0.36	0.45	0.63	0.84	0.88
RT-DETR	Level 1	0.99	0.89	0.78	0.49	0.51
	Level 2	0.88	0.96	0.88	0.6	0.51
	Level 3	0.48	0.79	0.89	0.81	0.6
	Level 4	0.4	0.4	0.56	0.62	0.6
	Level 5	0.49	0.46	0.7	0.75	0.78
YOLOv5	Level 1	0.92	0.75	0.5	0.49	0.45
	Level 2	0.85	0.88	0.84	0.7	0.51
	Level 3	0.52	0.72	0.87	0.73	0.61
	Level 4	0.4	0.42	0.61	0.66	0.59
	Level 5	0.3	0.54	0.65	0.7	0.72
YOLOv7	Level 1	0.98	0.9	0.85	0.72	0.71
	Level 2	0.83	0.93	0.79	0.69	0.61
	Level 3	0.4	0.72	0.95	0.75	0.74
	Level 4	0.41	0.52	0.69	0.87	0.84
	Level 5	0.42	0.52	0.55	0.74	0.9
Improved YOLOv8	Level 1	0.99	0.74	0.52	0.43	0.24
	Level 2	0.64	0.97	0.75	0.52	0.35
	Level 3	0.49	0.65	0.95	0.75	0.56
	Level 4	0.42	0.46	0.69	0.85	0.76
	Level 5	0.39	0.42	0.52	0.79	0.93

## Data Availability

The original contributions presented in this study are included in the article. Further inquiries can be directed to the corresponding author.

## Abbreviations

The following abbreviations are used in this manuscript:

YOLO	You Only Look Once
FPN	Feature Pyramid Network
PAN	Path Aggregation Network
CSPDarknet	Cross-Stage Partial Darknet
C2f	Faster Implementation of CSP Bottleneck with two convolutions
Conv	Convolution
SPPF	Spatial Pyramid Pooling Fast
mAP	Mean average precision
GELU	Gaussian Error Linear Units
SiLU	Sigmoid Linear Unit
RoI	Region of interest

## References

[1] Fang W., Chen H., Liu X. (2020). Research on Corrosion Causes of Common Metal Material in Power Grid.

[2] Landolfo R., Cascini L., Portioli F. (2010). Modeling of metal structure corrosion damage: A state of the art report. Sustainability.

[3] He Y., Song K., Meng Q., Yan Y. (2019). An end-to-end steel surface defect detection approach via fusing multiple hierarchical features. IEEE Trans. Instrum. Meas..

[4] Xu J., Gui C., Han Q. (2020). Recognition of rust grade and rust ratio of steel structures based on ensembled convolutional neural network. Comput. Aided Civ. Infrastruct. Eng..

[5] Nelson B.N., Slebodnick P.F., Lemieux E.J., Singleton W., Krupa M.S., Lucas K., Thomas E.D., Seelinger A. (2001). Wavelet processing for image denoising and edge detection in automatic corrosion detection algorithms used in shipboard ballast tank video inspection systems. Proceedings of the Wavelet Applications VIII.

[6] Liao K.W., Lee Y.T. (2016). Detection of rust defects on steel bridge coatings via digital image recognition. Autom. Constr..

[7] Tian Z., Zhang G., Liao Y., Li R., Huang F. (2019). Corrosion identification of fittings based on computer vision. Proceedings of the 2019 International Conference on Artificial Intelligence and Advanced Manufacturing (AIAM).

[8] Guo Z., Tian Y., Mao W. (2022). A robust faster R-CNN model with feature enhancement for rust detection of transmission line fitting. Sensors.

[9] Katsamenis I., Doulamis N., Doulamis A., Protopapadakis E., Voulodimos A. (2022). Simultaneous Precise Localization and Classification of metal rust defects for robotic-driven maintenance and prefabrication using residual attention U-Net. Autom. Constr..

[10] Zhao Z., Guo G., Zhang L., Li Y. (2023). A new anti-vibration hammer rust detection algorithm based on improved YOLOv7. Energy Rep..

[11] Wang Q., Wu B., Zhu P., Li P., Zuo W., Hu Q. ECA-Net: Efficient channel attention for deep convolutional neural networks. Proceedings of the IEEE/CVF Conference on Computer Vision and Pattern Recognition.

[12] Woo S., Park J., Lee J.Y., Kweon I.S. Cbam: Convolutional block attention module. Proceedings of the European Conference on Computer Vision (ECCV).

[13] Yu Q., Han Y., Lin W., Gao X. (2024). Detection and analysis of corrosion on coated metal surfaces using enhanced YOLO v5 algo rithm for anti-corrosion performance evaluation. J. Mar. Sci. Eng..

[14] Hussain M. (2023). YOLO-v1 to YOLO-v8, the rise of YOLO and its complementary nature toward digital manufacturing and industrial defect detection. Machines.

[15] Li C., Yan H., Qian X., Zhu S., Zhu P., Liao C., Tian H., Li X., Wang X., Li X. (2023). A domain adaptation YOLOv5 model for industrial defect inspection. Measurement.

[16] Wang Y., Wang H., Xin Z. (2022). Efficient detection model of steel strip surface defects based on YOLO-V7. IEEE Access.

[17] Zhao L., Li S. (2020). Object detection algorithm based on improved YOLOv3. Electronics.

[18] Sohan M., Sai R.T., Reddy R. (2024). A review on yolov8 and its advancements. Proceedings of the International Conference on Data Intelligence and Cognitive Informatics.

[19] Terven J., Córdova-Esparza D.M., Romero-González J.A. (2023). A comprehensive review of yolo architectures in computer vision: From yolov1 to yolov8 and yolo-nas. Mach. Learn. Knowl. Extr..

[20] Zhang X. (2024). Improved Multi-Detection Head Target Detection Algorithm For YOLOv8. Proceedings of the 2024 IEEE 2nd In ternational Conference on Image Processing and Computer Applications (ICIPCA).

[21] Zhang Y., Zhang H., Huang Q., Han Y., Zhao M. (2024). DsP-YOLO: An anchor-free network with DsPAN for small object detection of multiscale defects. Expert Syst. Appl..

[22] Xie Y., Shen J., Wu C. (2020). Affine geometrical region CNN for object tracking. IEEE Access.

[23] Nicheporuk A., Savenko O., Nicheporuk A., Nicheporuk Y. An Android Malware Detection Method Based on CNN Mixed Data Model. Proceedings of the ICTERI Workshops.

[24] Ding N., Möller K. (2023). The Image flip effect on a CNN model classification. Proc. Autom. Med. Eng..

[25] Zhang X., Chen Q., Ng R., Koltun V. Zoom to learn, learn to zoom. Proceedings of the IEEE/CVF Conference on Computer Vision and Pattern Recognition.

[26] Gao L., Wei X., Li C., Zhang X., Sun Y., Lin W. (2025). Threat target image detection method based on two-stage R-CNN network. Proceedings of the International Conference on Mechatronics and Intelligent Control (ICMIC 2024).

[27] Liu H., Qiao J., Li L., Wang L., Chu H., Wang Q. (2022). Parallel CNN Network Learning-Based Video Object Recognition for UAV Ground Detection. Wirel. Commun. Mob. Comput..

[28] Kim C., Shin D., Kim B., Park J. (2018). Mosaic-CNN: A combined two-step zero prediction approach to trade off accuracy and computation energy in convolutional neural networks. IEEE J. Emerg. Sel. Top. Circuits Syst..

[29] Wu Y., Liu L., Bae J., Chow K.-H., Iyengar A., Pu C., Wei W., Yu L., Zhang Q. (2019). Demystifying learning rate policies for high accuracy training of deep neural networks. Proceedings of the 2019 IEEE International Conference on Big Data (Big Data).

[30] Zhang C., Shao Y., Sun H., Xing L., Zhao Q., Zhang L. (2024). The WuC-Adam algorithm based on joint improvement of Warmup and cosine annealing algorithms. Math. Biosci. Eng.

[31] Altmayer F. (1985). Critical aspects of the salt spray test. Plat. Surf. Fin..

[32] Yakovlev A., Lisovychenko O. (2020). An approach for image annotation automatization for artificial intelligence models learning. Адаптивні системи автoматичнoгo управління.

[33] Vakili M., Ghamsari M., Rezaei M. (2020). Performance analysis and comparison of machine and deep learning algorithms for IoT data classification. arXiv.

[34] Ren S., He K., Girshick R., Sun J. (2016). Faster R-CNN: Towards real-time object detection with region proposal networks. IEEE Trans. Pattern Anal. Mach. Intell..

[35] Zhai S., Shang D., Wang S., Dong S. (2020). DF-SSD: An improved SSD object detection algorithm based on DenseNet and feature fusion. IEEE Access.

[36] Zhu M., Kong E. (2024). Multi-scale fusion uncrewed aerial vehicle detection based on RT-DETR. Electronics.

[37] Olorunshola O.E., Irhebhude M.E., Evwiekpaefe A.E. (2023). A comparative study of YOLOv5 and YOLOv7 object detection algorithms. J. Comput. Soc. Inform..

